# Temporal trends in post-extubation respiratory management and reintubation risk factors in Japan: A retrospective multicenter cohort study

**DOI:** 10.1016/j.ccrj.2026.100170

**Published:** 2026-02-25

**Authors:** Toshinori Maezawa, Masaaki Sakuraya, Akihiro Takaba

**Affiliations:** a Department of Emergency and Intensive Care Medicine, JA Hiroshima General Hospital, 1-3-3 Jigozen, Hatsukaichi, Hiroshima, 738-8503, Japan; b Graduate School of Data Science, Shiga University, 1-1-1 Banba, Hikone, Shiga 522-8522, Japan

**Keywords:** Airway extubation, Critical care, Noninvasive ventilation, Respiration, Artificial, Ventilator weaning

## Abstract

**Objective:**

Non-invasive ventilation (NIV) and high-flow nasal cannula (HFNC) have been used to prevent reintubation. We aimed to describe the utilisation patterns and analyze temporal trends of NIV and HFNC after extubation.

**Design:**

Retrospective multicenter cohort study using the Japanese Intensive care PAtient Database (JIPAD) from 2018 to 2022.

**Setting:**

Facilities that consecutively registered cases in JIPAD during the study period.

**Participants:**

We included adult patients (>18 years) who were receiving mechanical ventilation at the time of intensive care unit (ICU) admission, with a duration of mechanical ventilation of at least 24 h.

**Interventions:**

None.

**Main outcome measures:**

Temporal trends in the utilisation of NIV and HFNC after extubation over the 5-year study period.

**Results:**

We included 12,687 eligible patients from 40 ICUs. Based on the Cochran–Armitage test, the proportion of patients receiving NIV decreased from the years 2018 to 2022 (6.7-3.9 %, P for trend <0.001), while that receiving HFNC significantly increased (15.9-28.0 %, P for trend <0.001). After multivariable adjustment (with 2018 as the reference year) and relative to oxygen therapy, the year 2022 was associated with a significant decrease in NIV (adjusted odds ratio, 0.67; 95 % confidence interval, 0.52-0.88) and a significant increase in HFNC (adjusted odds ratio, 1.89; 95 % confidence interval, 1.62-2.21).

**Conclusions:**

We analysed over 12,000 patients in this retrospective multicenter cohort study. The proportion of HFNC use after extubation increased, while NIV use decreased, and these changes remained significant after multivariable analysis. Further research is warranted to clarify appropriate indications for NIV and HFNC after extubation.

## 1 Introduction

Strategies to facilitate successful liberation from mechanical ventilation are important because extubation failure is associated with an increased risk of hospital mortality.^1^^,^^2^ To prevent reintubation, non-invasive ventilation (NIV) and high-flow nasal cannula (HFNC) are commonly employed after extubation. Clinical practice guidelines primarily recommend the prophylactic use of NIV and HFNC for patients with any reintubation risk factors (e.g. older age, obesity, heart failure).[3], [4], [5]

The use of NIV and HFNC after extubation is expected to increase along with guideline recommendations and the accumulation of evidence.[6], [7], [8], [9], [10], [11], [12], [13] Traditionally, NIV has been indicated for patients with specific conditions including heart failure or hypercapnia. Subsequently, HFNC has been widely adopted as an alternative, having demonstrated non-inferiority to NIV^15^^,^^16^ and greater patient comfort.^17^^,^^18^ Although the optimal strategy for selecting between these two modalities is not yet established, NIV tends to be preferred for patients with a higher number of reintubation risk factors (e.g. four or more).^13^^,^^14^ However, the actual utilisation patterns and trends of post-extubation NIV and HFNC in clinical practice remain unclear. In addition, to interpret these trends, it is also important to consider the prevalence of reintubation risk factors. While previous studies have determined the effect sizes of individual risk factors for reintubation,^19^ few have examined their actual prevalence and distribution in a real-world setting.

The primary objective of this study was to describe the utilisation patterns and analyse the temporal trends of post-extubation NIV and HFNC over a 5-year period, with a secondary objective of characterising the epidemiology of reintubation risk factors. Therefore, we performed a retrospective multicenter cohort study using a Japanese intensive care unit (ICU) database.

## 2 Methods

### 2.1 Study design and data

This retrospective cohort study was performed using data from the Japanese Intensive care PAtient Database (JIPAD). The JIPAD is a nationwide, multicenter observational data registry for critically ill patients that was established in 2014 by the Japanese Society of Intensive Care Medicine and modelled after the Australian and New Zealand Intensive Care Society Adult Patient Database.^20^^,^^21^ With the aim of improving quality of care and developing intensive care practices, JIPAD collects clinical data, including patient demographics, diagnosis at ICU admission, physiological data and treatment in the ICU, and patient outcomes. As of February 2024, 118 ICUs had participated in this registry, representing approximately one-sixth of the ICUs in Japan.

The present study was reported according to the Strengthening the Reporting of OBservational studies in Epidemiology.^22^ The study protocol was approved by the Ethics Review Board of JA Hiroshima General Hospital (approval number: 23-23), with a waiver for written consent for participation.

### 2.2 Study population

From April 2018 to March 2023, we included adult patients (>18 years) who were receiving mechanical ventilation at the time of admission to eligible ICUs, with a duration of mechanical ventilation of at least 24 h to exclude routine postoperative monitoring, consistent with previous studies.^8^^,^^9^ We selected this timeframe because JIPAD initiated data collection on NIV and HFNC use, as well as reintubation events, in April 2018. Eligible ICUs were defined as those that continuously registered patients in the JIPAD registry during the study period. We excluded patients based on the following criteria: 1) those with a tracheostomy on ICU admission, 2) those who underwent tracheostomy prior to the first extubation attempt, 3) those who did not undergo extubation during their ICU stay (including those who died), and 4) those with missing data on NIV and HFNC use and reintubation risk factors.

### 2.3 Data collection

The following data were collected: age, sex, body mass index (BMI), comorbidities (chronic heart failure, chronic respiratory failure, liver cirrhosis, haematological malignancy, metastatic cancer, immunosuppression, and maintenance dialysis [yes, no] (Appendix 1 in Additional file)), type of ICU admission (medical, elective surgery, or emergency surgery), tracheostomy on ICU admission, Acute Physiology And Chronic Health Evaluation (APACHE) scores,^23^ Sequential Organ Failure Assessment (SOFA) scores,^24^ NIV [yes, no], HFNC [yes, no], the duration of mechanical ventilation (recorded for each period from intubation to extubation), the lengths of ICU and hospital stays, ICU and hospital mortality [yes, no], tracheostomy [yes, no], and the day of tracheostomy. The presence of COVID-19 was defined by whether it was the primary disease at the time of ICU admission. Clinical outcomes were the standardised mortality ratio calculated with the APACHE III score, ventilator-free days, length of ICU stay, and the reintubation rate for each fiscal year. Reintubation was defined as the re-initiation of mechanical ventilation within 72 h after the first extubation.^6^^,^^7^^,^^12^

### 2.4 Risk factors for reintubation and post-extubation respiratory management

In the present study, the following six factors were defined as risk factors for reintubation based on a priori knowledge[7], [8], [9]: 1) age ≥65 years, 2) BMI ≥30 kg/m^2^, 3) heart failure, 4) chronic obstructive pulmonary disease, 5) two or more comorbidities, and 6) duration of mechanical ventilation >7 days (detailed definitions are provided in Appendix 2 in the Additional file). We classified post-extubation respiratory management into four mutually exclusive strategies based on the combination of recorded responses (“yes” or “no”) for NIV and HFNC usage: oxygen therapy (NIV: no/HFNC: no), NIV alone (NIV: yes/HFNC: no), HFNC alone (NIV: no/HFNC: yes), and NIV + HFNC (NIV: yes/HFNC: yes).

### 2.5 Statistical analysis

Continuous variables were described as medians and interquartile ranges (IQRs), while categorical variables were presented as numbers and percentages (%). To validate our risk stratification approach in our cohort, we assessed the relationship between the number of accumulated reintubation risk factors (0, 1, 2, or ≥3) and the proportion of reintubation, consistent with previous findings.^14^ Due to dataset specifications, we could not definitively distinguish between the prophylactic and therapeutic use of NIV or HFNC. To evaluate the clinical context, we analysed the distribution of time from initial extubation to ICU discharge for each strategy.

Our primary analysis focused on the temporal trends of post-extubation respiratory management over the 5-year period (2018-2022). The study year corresponded to the Japanese fiscal year, which extends from April 1 to March 31 of the following year. First, we assessed the temporal trends in the annual proportion of each strategy (oxygen therapy, NIV, HFNC, and NIV + HFNC), using the Cochran–Armitage test. Second, to determine the adjusted temporal trends, a multivariable multinomial logistic analysis using a mixed-effects model was conducted. The dependent variable was the choice of post-extubation respiratory strategy, a four-level categorical variable, with oxygen therapy serving as the reference category. The variables for fixed effects were the risk factors (older age, BMI, heart failure, chronic obstructive pulmonary disease, two or more comorbidities, and prolonged mechanical ventilation), fiscal year (with 2018 as the reference category), admission type (emergency and surgery), and the APACHE II score at ICU admission. To adjust for potential differences in clinical practice among facilities, a random effect was included for each participating facility. This variation among facilities was also visualised. Furthermore, we conducted subgroup analyses to evaluate the trends in post-extubation respiratory management stratified by the type of ICU admission (medical, elective surgery, or emergency surgery), the number of reintubation risk factors (0, 1, or ≥2), and specific risk factors (prolonged mechanical ventilation, heart failure, and obesity [BMI ≥30 kg/m^2^]).

Results are presented as odds ratios (ORs) with 95 % confidence intervals (CIs). All statistical tests were two-sided, and P values < 0.05 were considered statistically significant. For subgroup analyses, we applied the Bonferroni correction to account for multiple comparisons. When the proportion of missing data was less than 5 % for all covariates, we performed a complete case analysis. All statistical analyses were performed using R (2024, version 4.3.3; The R Foundation for Statistical Computing, Vienna, Austria).

## 3 Results

### 3.1 Study participants and patient characteristics

During the study period, 276,192 patients were enrolled in the JIPAD registry, and 40 of the 95 facilities consecutively registered cases (Supplemental Figure 1). A total of 169,234 patients were admitted to the 40 eligible ICUs, and according to inclusion and exclusion criteria, 12,687 ICU patients were included in this study.

The median age of patients was 71 (interquartile range [IQR], 59-78) years, and 4476 (35.3 %) were female (Table 1). The median BMI was 22.9 (IQR, 20.2-25.8) kg/m^2^. The median SOFA score was 8 (IQR, 6-10) and the median APACHE II score was 19 (IQR, 15-24). While the reintubation rate decreased from 12.8 % in 2018 to 5.9 % in 2022, the standardised mortality ratio remained stable throughout the study period (Table 1).

**Table 1:** Baseline characteristics and clinical outcomes.

	All	Fiscal year a				
		2018	2019	2020	2021	2022
	(n = 12,687)	(n = 2244)	(n = 2453)	(n = 2550)	(n = 2735)	(n = 2705)
Age, median (IQR)	71 (59-78)	70 (60-78)	70 (59-79)	70 (59-78)	71 (57-78)	72 (59-79)
Female, n (%)	4476 (35.3)	844 (37.6)	899 (36.6)	871 (34.2)	904 (33.1)	958 (35.4)
BMI, kg/m^2^, median (IQR)	22.9 (20.2-25.8)	22.4 (20.0-25.2)	22.9 (20.1-25.7)	23.0 (20.3-26.1)	23.2 (20.4-26.1)	23.0 (20.3-25.9)
COVID-19, n (%)	318 (3.4)	0 (0.0)	3 (0.2)	97 (5.0)	167 (7.8)	51 (2.7)
Type of ICU admission, n (%)						
Medical	4606 (36.3)	748 (33.3)	863 (35.2)	995 (39.0)	1119 (40.9)	881 (32.6)
Elective surgery	3751 (29.6)	739 (32.9)	696 (28.4)	729 (28.6)	725 (26.5)	862 (31.9)
Emergency surgery	4330 (34.1)	757 (33.7)	894 (36.4)	826 (32.4)	891 (32.6)	962 (35.6)
SOFA, median (IQR)	8 (7-10)	8 (7-11)	9 (7-11)	8 (6-10)	8 (6-10)	8 (7-10)
APACHE II, median (IQR)	19 (15-24)	19 (15-24)	19 (16-25)	19 (15-25)	19 (15-24)	19 (16-24)
APACHE III, median (IQR)	74 (60-92)	75 (62-92)	75 (60-93)	74 (59-94)	72 (58-90)	74 (60-92)
Details of reintubation risk factors, n (%)						
Age >65 years	8348 (65.8)	1512 (67.4)	1616 (65.9)	1667 (65.4)	1731 (63.3)	1822 (67.4)
BMI >30 kg/m^2^	981 (7.7)	139 (6.2)	166 (6.8)	220 (8.6)	237 (8.7)	219 (8.1)
Heart failure	1423 (11.2)	243 (10.8)	272 (11.1)	290 (11.4)	317 (11.6)	301 (11.1)
COPD	235 (1.9)	47 (2.1)	50 (2.0)	37 (1.5)	47 (1.7)	54 (2.0)
Two or more comorbidities b	266 (2.1)	47 (2.1)	52 (2.1)	53 (2.1)	57 (2.1)	57 (2.1)
Prolonged mechanical ventilation c	2336 (18.4)	357 (15.9)	460 (18.8)	487 (19.1)	525 (19.2)	507 (18.7)
SMR d	0.326	0.301	0.324	0.339	0.312	0.349
ICU days, day (median [IQR])	7 (5-11)	7 (5-10)	7 (5-11)	7 (5-11)	7 (5-11)	7 (5-11)
Ventilator-free-days, day (median [IQR])	24 (20-25)	24 (21-25)	24 (20-25)	23 (20-25)	23 (20-25)	24 (20-25)
Reintubation within 72 h	1013 (8.0)	288 (12.8)	179 (7.3)	188 (7.4)	198 (7.2)	160 (5.9)

APACHE, Acute Physiology And Chronic Health Evaluation; BMI, body mass index; COPD, chronic obstructive pulmonary disease; ICU, intensive care unit; IQR, interquartile range; MV, mechanical ventilation; SMR, standardised mortality ratio; SOFA, Sequential Organ Failure Assessment.

a Each fiscal year extended from April 1 to March 31 of the following year.

b Definitions of each comorbidity are summarised in Supplementary Appendix 1.

c Prolonged mechanical ventilation is defined as a patient receiving mechanical ventilation for more than seven days.

d SMR was calculated as the sum of observed deaths divided by the sum of predicted mortality based on the APACHE III score.

### 3.2 Prevalence of risk factors for reintubation

In the present study, the distribution of reintubation risk factors is shown in Supplemental Table 1. There were 2677 patients with no identified risk factors (21.1 %) in the cohort, while 3047 (24.0 %) patients had two or more risk factors. Reintubation rates significantly increased with the number of risk factors (Supplemental Figure 2): 4.2 % for no risk factors, 6.7 % for one, 13.8 % for two, and 15.9 % for three or more.

### 3.3 Overview of post-extubation respiratory management

Eligible patients received post-extubation respiratory management as follows: oxygen therapy (n = 8,614, 67.9 %), NIV (n = 717, 5.7 %), HFNC (n = 2,625, 20.7 %), and NIV + HFNC (n = 731, 5.8 %), after excluding a small number of cases with missing data on NIV/HFNC usage (n = 5) or BMI (n = 132). At ICU admission, no clinically relevant differences were observed in APACHE II and SOFA scores among the respiratory management (Supplemental Figure 3). The distribution of post-extubation respiratory strategies varied across facilities, ranging from 89.7 % at the facility with the highest usage to 7.2 % at the facility with the lowest usage (Supplemental Figure 4). Approximately half of the patients in the NIV and HFNC groups were discharged from the ICU within 72 h (NIV: 49.5 % and HFNC: 52.3 %), compared to only 23.7 % in the NIV + HFNC group (Supplementary Figure 5).

### 3.4 Temporal trends in strategy selection

**Fig. 1:**
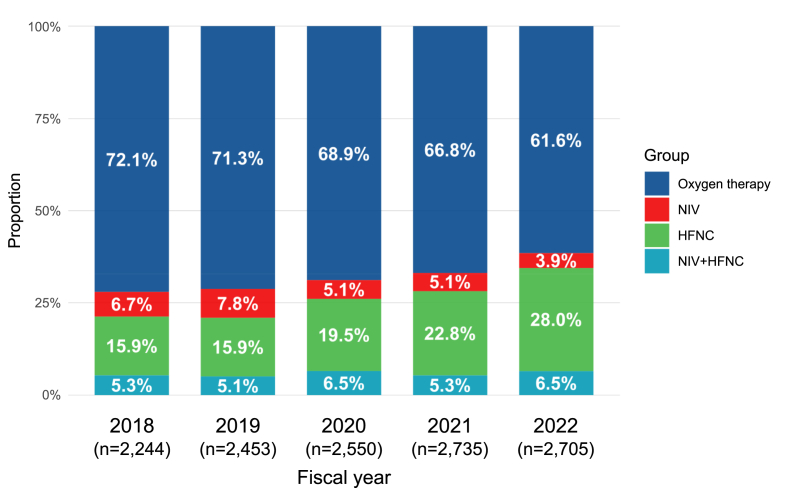
Temporal trends in the proportion of each respiratory management from 2018 to 2022. Oxygen therapy was defined as the absence of both NIV and HFNC usage. P for trend: Oxygen therapy, P < 0.001; NIV, P < 0.001; HFNC, P < 0.001; and NIV + HFNC, P = 0.088. HFNC, high-flow nasal cannula; NIV, non-invasive ventilation.

Although the proportion of patients receiving oxygen therapy significantly decreased from 72.1 % in 2018 to 61.6 % in 2022 (P for trend <0.001), it remained consistently above 60 % throughout the study period (Fig. 1). In contrast, the use of HFNC significantly increased from 15.9 to 28.0 % (P for trend <0.001). A decrease was also observed for NIV (from 6.7 to 3.9 %, P for trend <0.001). A consistent trend was observed across many subgroups (Supplemental Figure 6 and Supplemental Table 2).

**Fig. 2:**
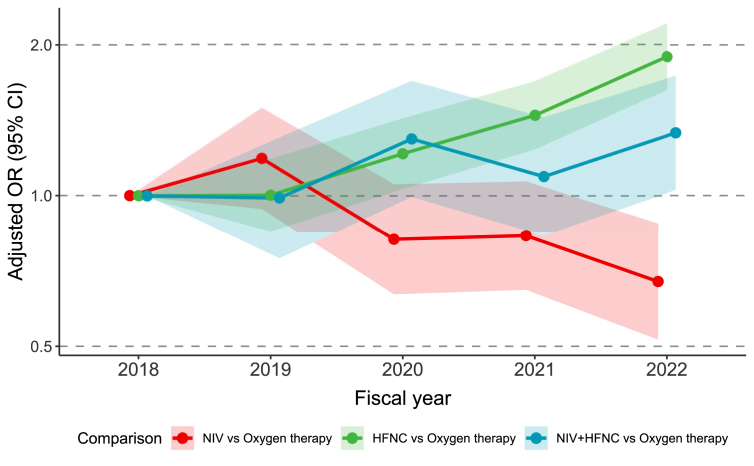
Adjusted odds ratio for the selection of post-extubation respiratory strategy by fiscal year. Adjusted odds ratios (ORs) with 95 % confidence intervals (CIs) for the selection of NIV and HFNC strategy compared to oxygen therapy, by fiscal year. ORs were estimated from a multivariable multinomial logistic regression analysis, adjusted for the reintubation risk factors, admission type, and the APACHE II score at ICU admission, with participating facilities included as a random effect; 2018 served as the reference year. Each fiscal year extended from April 1 to March 31 of the following year. APACHE, Acute Physiology And Chronic Health Evaluation; ICU, intensive care unit; CI, confidence interval; HFNC, high-flow nasal cannula; NIV, non-invasive ventilation.

After adjusting for reintubation risk factors and other covariates, the temporal trends in the selection of post-extubation respiratory strategy compared to oxygen therapy were analysed (Fig. 2). The likelihood of selecting NIV significantly decreased over the study period; compared to 2018, the adjusted OR for NIV selection in 2022 was 0.67 (95 % CI, 0.52-0.88) (Supplemental Table 3). Conversely, the use of HFNC showed a significant increase in trends, with the adjusted OR for its selection in 2022 being 1.89 (95 % CI, 1.62-2.21) compared to 2018.

## 4 Discussion

The present study described the utilisation patterns and analysed temporal trends of post-extubation respiratory management in Japanese ICUs from 2018 to 2022. We included more than 12,000 mechanically ventilated patients in 40 ICUs, noting that nearly 80 % had at least one risk factor. During the study period, the use of HFNC significantly increased from 15.9 to 28.0 %, while that of NIV and oxygen therapy decreased. Even after adjusting for covariates, over the 5-year study period, HFNC use showed a significant increasing trend, whereas NIV use showed a significant decreasing trend.

Previous studies that compared the effects of post-extubation respiratory support showed that the proportion of patients at risk of reintubation widely varied between 42 and 70 %.[7], [8], [9]^,^^12^ In our study, nearly 80 % of patients had at least one risk factor, while 24.0 % had multiple risk factors. The most common risk factor was older age (69.0 %; median, 72 years), and the prevalence of elderly patients was similar to that in previous studies. In contrast, other risk factors, including prolonged mechanical ventilation (19.7 %), heart failure (12.1 %), and obesity (9.4 %), were less frequent. This discrepancy may be attributed to a selection bias; elderly patients with multiple comorbidities may have been excluded from our study population due to do-not-intubate orders.^25^

In clinical practice guidelines,^3^^,^^4^ the recommendation for NIV as post-extubation respiratory support was established in 2017 and that for HFNC in 2020. Since NIV and HFNC are recommended over oxygen therapy for patients with at least one risk factor for reintubation,[3], [4], [5] these supports were indicated for many patients after extubation. The present study showed that the use of NIV and HFNC after extubation significantly increased by more than 10 %, from 27.9 % in 2018 to 38.4 % in 2022. This increasing adoption of NIV and HFNC suggests a move towards more evidence-based post-extubation respiratory management. Consistent with this trend, the reintubation rate in our cohort peaked in 2018 and subsequently decreased. Given that patient characteristics remained stable, this improvement might be associated with the expanded use of NIV and HFNC. However, caution is warranted regarding causality. Unmeasured confounders, including changes in patient code status or the inability to distinguish between prophylactic and therapeutic use, exist in this database study; therefore, a definitive causal relationship cannot be established.

The primary finding of this study was a marked temporal shift in device selection, characterised by a decrease in NIV use and an increase in HFNC use. These trends remained significant after adjusting for various covariates and was consistent across many subgroups. This shift likely reflects the increasing prioritisation of HFNC over NIV by ICU physicians, driven by its advantages in patient comfort and accumulating evidence supporting its non-inferiority to NIV for preventing treatment failure.^7^^,^^15^ From a resource perspective, the shift from relatively low-cost oxygen therapy to NIV and HFNC is associated with not only higher financial costs but also an increased nursing workload. Therefore, rather than promoting generalised adoption, further research is warranted to precisely identify the patient populations that would truly benefit from NIV and HFNC.

To the best of our knowledge, this is the first epidemiological study to use real-world data to describe the utilisation patterns and analyze the trends of post-extubated respiratory management. These results have important clinical implications for the current decision-making process in post-extubation respiratory management. Specifically, there is a need to re-evaluate how risk factors are used to guide NIV and HFNC selection. Despite the overall increase in NIV and HFNC use, more than half of patients with two or more risk factors received neither NIV nor HFNC. This suggests that ICU physicians may consider NIV and HFNC unnecessary for many patients based on their bedside assessment. This implies a discrepancy with conventional criteria, which rely simply on the number of reintubation risk factors. This gap may exist because reintubation risk factors primarily predict extubation failure, rather than directly guiding the optimal selection of NIV or HFNC. Therefore, future studies need to investigate how not only the number of risk factors but also their specific types, characteristics, and relative weighting, are associated with differences in the efficacy of post-extubation respiratory strategy.

The present study has several limitations that need to be addressed. First, the retrospective observational design of this study does not allow for any conclusions about causality. Specifically, due to the nature of the database, we could not access detailed device settings (*e.g.*NIV interfaces) or granular clinical factors crucial for extubation decisions, such as patient code status (e.g. do not reintubate), sputum volume, or cough strength. Since these unmeasured variables act as major confounders in the relationship between device selection and extubation outcomes, we could not evaluate the comparative efficacy of these devices. Consequently, our analysis was specifically designed to investigate the association between fiscal years and device selection patterns. Second, the external validity of our findings is limited regarding both the study setting and its timing. Since we analysed data exclusively from Japanese ICUs between 2018 and 2022, caution is warranted when generalising these results to other countries or different timeframes. Regarding the setting, factors specific to the Japanese population may have influenced the results. For example, the increasing HFNC use might reflect the priority placed on comfort given the high prevalence of elderly patients, while the decreasing trend in NIV could be influenced by the relatively low prevalence of obesity. The fundamental clinical reasoning behind these practice patterns is likely universal. Regarding the timing, the study period overlapped the COVID-19 pandemic, which may have uniquely affected clinical practice. However, the observed trends persisted through fiscal year 2022, a period when the pandemic’s influence was diminishing. This suggests that the shifts in respiratory management identified in our study are not merely transient but likely represent sustained changes.

## 5 Conclusion

From 2018 to 2022, the use of HFNC after extubation significantly increased, while that of NIV decreased. This change remained significant even after adjusting for covariates. Nearly 80 % of patients who received mechanical ventilation had at least one risk factor for reintubation. Further research is warranted to clarify appropriate indications for NIV and HFNC after extubation based on patient risk factors.

## Financial support

This research did not receive any specific grant from funding agencies in the public, commercial, or not-for-profit sectors.

## Declaration of competing interest

The authors declare that they have no known competing financial interests or personal relationships that could have appeared to influence the work reported in this paper.
