# Supplementary material for: Temporal trends in post-extubation respiratory management and reintubation risk factors in Japan: A retrospective multicenter cohort study
Source: Crit Care Resusc. 2026 Feb 25;28(1):100170. doi: 10.1016/j.ccrj.2026.100170 (PMC12955550; doi:10.1016/j.ccrj.2026.100170)
Supplement: Multimedia component 1 [file mmc1.pdf]

Supplemental Appendix 1. Criteria for comorbidities in the Japanese Intensive care PAtient Database (JIPAD)

- **Chronic heart failure**

  NYHA Class IV (symptoms at rest or with minimal exertion)

- **Chronic respiratory failure**

  Proven chronic hypoxemia, hypercapnia, secondary polycythemia, severe pulmonary hypertension, or ventilator dependence

- **Liver cirrhosis**

  Underlying cause of liver cirrhosis with signs of portal hypertension (e.g., esophageal varices) or confirmed by biopsy

- **Hematological malignancy**

  History within the past 5 years of leukemia (acute/chronic, myeloid/lymphoid) or multiple myeloma; or history within the past 5 years of lymphoma

- **Metastatic cancer**

  Presence of distant metastasis from solid cancer (excluding regional lymph node involvement or invasion); peritoneal dissemination is considered to be distant metastasis

- **Immunosuppression**

  AIDS (HIV-positive with AIDS-defining complications) or immunosuppression (received immunosuppressants, chemotherapy, radiation therapy, or steroids (prednisolone equivalent $\geq$ 0.375 mg/kg/day) within the past 6 months)

- **Maintenance dialysis**

  On hemodialysis or peritoneal dialysis for at least 3 months

Supplemental Appendix 2. Criteria for reintubation risk factors

We identified six risk factors for reintubation extractable from the JIPAD, based on criteria used in previous randomized clinical trials [1-3]. The definitions adopted in this study are as follows:

1) Age ≥65 years

Defined as age at hospital admission being 65 years or older.

2) Obesity (BMI ≥30 kg/m²)

Defined as a body mass index (BMI) of 30 kg/m² or greater, calculated based on height and weight recorded at ICU admission.

3) Heart failure

Unlike previous studies that defined this variable based on the primary reason for mechanical ventilation, we defined it by the presence of primary disease codes associated with cardiac dysfunction at ICU admission. These included cardiogenic shock, cardiac arrest, congestive heart failure, arrhythmia, acute coronary syndrome, hypertensive emergency, cardiomyopathy, and unstable angina.

4) Chronic obstructive pulmonary disease (COPD)

While previous studies defined this variable as a history of moderate-to-severe COPD, we defined this factor as having either a primary diagnosis of COPD exacerbation or a documented comorbidity of chronic respiratory failure at ICU admission.

5) Two or more comorbidities

Defined as the presence of two or more comorbidities listed in Appendix 1.

6) Prolonged mechanical ventilation

Defined as a duration of the first mechanical ventilation episode exceeding 7 days.

Reference:

1) Hernández G, et al. Effect of postextubation high-flownasal cannula vs noninvasive ventilation on reintubation and postextubation respiratory failure in high-risk patients a randomized clinical trial. JAMA 2016;316:1565–1574

2) Thille AW, et al. Effect of Postextubation High-Flow Nasal Oxygen with Noninvasive Ventilation vs High-Flow Nasal Oxygen Alone on Reintubation among Patients at High Risk of Extubation Failure: A Randomized Clinical Trial. JAMA 2019; 322: 1465–75.

3) Hernández G, et al. Effect of postextubation noninvasive ventilation with active humidification vs high-flow nasal cannula on reintubation in patients at very high risk for extubation failure: a randomized trial. Intensive Care Med 2022; 48: 1751–9.

Supplemental Table 1 Prevalence of comorbidities and distribution of the number of risk factors

	All	Fiscal year [a]				
	(n = 12,687)	2018 (n = 2,244)	2019 (n = 2,453)	2020 (n = 2,550)	2021 (n = 2,735)	2022 (n = 2,705)
Comorbidities [b], n (%)						
Chronic heart failure	337 (2.7)	53 (2.4)	76 (3.1)	67 (2.6)	72 (2.6)	69 (2.6)
Chronic respiratory failure	204 (1.6)	35 (1.6)	42 (1.7)	34 (1.3)	44 (1.6)	49 (1.8)
Liver cirrhosis	173 (1.4)	27 (1.2)	35 (1.4)	39 (1.5)	37 (1.4)	35 (1.3)
Hematological malignancy	125 (1.0)	19 (0.8)	17 (0.7)	29 (1.1)	28 (1.0)	32 (1.2)
Metastatic cancer	256 (2.0)	58 (2.6)	53 (2.2)	45 (1.8)	47 (1.7)	53 (2.0)
Immunosuppression	553 (4.4)	112 (5.0)	125 (5.1)	101 (4.0)	115 (4.2)	100 (3.7)
Maintenance dialysis	815 (6.4)	133 (5.9)	175 (7.1)	164 (6.4)	176 (6.4)	167 (6.2)
Number of risk factors, n (%)						
0	2677 (21.1)	468 (20.9)	519 (21.2)	527 (20.7)	618 (22.6)	545 (20.1)
1	6963 (54.9)	1290 (57.5)	1354 (55.2)	1377 (54.0)	1454 (53.2)	1488 (55.0)
2	2556 (20.1)	411 (18.3)	487 (19.9)	568 (22.3)	538 (19.7)	552 (20.4)
3 or more	491 (3.9)	75 (3.3)	93 (3.8)	78 (3.1)	125 (4.6)	120 (4.4)

[a] Each fiscal year extended from April 1 to March 31 of the following year.

[b] Detailed definitions of each comorbidity are provided in Appendix 1

Supplemental Table 2. Proportions and trends of post-extubation respiratory management in patient subgroups

Fiscal year [a]	2018		2019		2020		2021		2022		P value [b]
(A) Type of ICU admission, n (%)											
Medical	748		863		995		1119		881		
Oxygen therapy	554	(74.1)	647	(75.0)	749	(75.3)	788	(70.4)	598	(67.9)	< 0.001
NIV	55	(7.4)	82	(9.5)	51	(5.1)	53	(4.7)	35	(4.0)	< 0.001
HFNC	107	(14.3)	101	(11.7)	160	(16.1)	243	(21.7)	197	(22.4)	< 0.001
NIV+HFNC	32	(4.3)	33	(3.8)	35	(3.5)	35	(3.1)	51	(5.8)	0.295
Elective surgery	739		696		729		725		862		
Oxygen therapy	508	(68.7)	440	(63.2)	442	(60.6)	430	(59.3)	472	(54.8)	< 0.001
NIV	55	(7.4)	57	(8.2)	38	(5.2)	49	(6.8)	39	(4.5)	0.008
HFNC	127	(17.2)	140	(20.1)	172	(23.6)	176	(24.3)	279	(32.4)	< 0.001
NIV+HFNC	49	(6.6)	59	(8.5)	77	(10.6)	70	(9.7)	72	(8.4)	0.185
Emergency surgery	757		894		826		891		962		
Oxygen therapy	555	(73.3)	661	(73.9)	565	(68.4)	609	(68.4)	596	(62.0)	< 0.001
NIV	40	(5.3)	53	(5.9)	41	(5.0)	37	(4.2)	32	(3.3)	0.009
HFNC	123	(16.2)	148	(16.6)	166	(20.1)	204	(22.9)	282	(29.3)	< 0.001
NIV+HFNC	39	(5.2)	32	(3.6)	54	(6.5)	41	(4.6)	52	(5.4)	0.475

Fiscal year [a]	**2018**		**2019**		**2020**		**2021**		**2022**		**P value [b]**
(B) Number of risk factors, n (%)											
No risk factor	468		519		527		618		545		
Oxygen therapy	377	(80.6)	411	(79.2)	405	(76.9)	454	(73.5)	392	(71.9)	< 0.001
NIV	25	(5.3)	27	(5.2)	14	(2.7)	27	(4.4)	15	(2.8)	0.033
HFNC	54	(11.5)	61	(11.8)	87	(16.5)	112	(18.1)	121	(22.2)	< 0.001
NIV+HFNC	12	(2.6)	20	(3.9)	21	(4.0)	25	(4.0)	17	(3.1)	0.651
One risk factor	1290		1354		1377		1454		1488		
Oxygen therapy	954	(74.0)	979	(72.3)	938	(68.1)	975	(67.1)	888	(59.7)	< 0.001
NIV	78	(6.0)	87	(6.4)	71	(5.2)	59	(4.1)	57	(3.8)	< 0.001
HFNC	199	(15.4)	225	(16.6)	285	(20.7)	343	(23.6)	447	(30.0)	< 0.001
NIV+HFNC	59	(4.6)	63	(4.7)	83	(6.0)	77	(5.3)	96	(6.5)	0.022
Two or more risk factors	411		487		568		538		552		
Oxygen therapy	239	(58.2)	308	(63.2)	364	(64.1)	327	(60.8)	319	(57.8)	0.482
NIV	41	(10.0)	65	(13.3)	41	(7.2)	40	(7.4)	30	(5.4)	< 0.001
HFNC	87	(21.2)	83	(17.0)	111	(19.5)	138	(25.7)	156	(28.3)	< 0.001
NIV+HFNC	44	(10.7)	31	(6.4)	52	(9.2)	33	(6.1)	47	(8.5)	0.317

Fiscal year [a]	2018		2019		2020		2021		2022		P value [b]
(C) Specific risk factors, n (%)											
Prolonged mechanical ventilation	357		460		487		525		507		
Oxygen therapy	210	(58.8)	272	(59.1)	294	(60.4)	304	(57.9)	265	(52.3)	0.037
NIV	21	(5.9)	51	(11.1)	28	(5.7)	29	(5.5)	22	(4.3)	0.007
HFNC	86	(24.1)	96	(20.9)	100	(20.5)	150	(28.6)	169	(33.3)	< 0.001
NIV+HFNC	40	(11.2)	41	(8.9)	65	(13.3)	42	(8.0)	51	(10.1)	0.475
Heart failure	243		272		290		317		301		
Oxygen therapy	169	(69.5)	195	(71.7)	219	(75.5)	222	(70.0)	207	(68.8)	0.628
NIV	25	(10.3)	41	(15.1)	21	(7.2)	27	(8.5)	15	(5.0)	0.001
HFNC	36	(14.8)	25	(9.2)	39	(13.4)	54	(17.0)	57	(18.9)	0.011
NIV+HFNC	13	(5.3)	11	(4.0)	11	(3.8)	14	(4.4)	22	(7.3)	0.256
Obesity (BMI ≥30kg/m^2)	139		166		220		237		219		
Oxygen therapy	76	(54.7)	105	(63.3)	131	(59.5)	118	(49.8)	109	(49.8)	0.022
NIV	19	(13.7)	17	(10.2)	21	(9.5)	24	(10.1)	17	(7.8)	0.113
HFNC	36	(25.9)	34	(20.5)	49	(22.3)	76	(32.1)	70	(32.0)	0.011
NIV+HFNC	8	(5.8)	10	(6.0)	19	(8.6)	19	(8.0)	23	(10.5)	0.076

BMI, body mass index; HFNC, high-flow nasal cannula; ICU, intensive care unit; NIV, non-invasive ventilation

[a] Each fiscal year extended from April 1 to March 31 of the following year.

[b] The significance threshold was adjusted to P <0.017 using the Bonferroni correction to account for the analyses performed within each of the three subgroups (A, B, and C).

Supplemental Table 3. Multivariable analysis of factors associated with post-extubation respiratory management selection

Variables	Adjusted OR (95% CI)	P value
NIV~2019	1.19 (0.94–1.50)	0.147
HFNC~2019	1.00 (0.85–1.19)	0.970
NIV+HFNC~2019	0.99 (0.75–1.31)	0.945
NIV~2020	0.82 (0.64–1.06)	0.122
HFNC~2020	1.21 (1.03–1.43)	0.019
NIV+HFNC~2020	1.30 (0.99–1.69)	0.055
NIV~2021	0.83 (0.65–1.07)	0.150
HFNC~2021	1.45 (1.24–1.69)	< 0.001
NIV+HFNC~2021	1.09 (0.83–1.43)	0.524
NIV~2022	0.67 (0.52–0.88)	0.004
HFNC~2022	1.89 (1.62–2.21)	< 0.001
NIV+HFNC~2022	1.34 (1.03–1.74)	0.031

CI, confidence interval; HFNC, high-flow nasal cannula; NIV, non-invasive ventilation; OR, odds ratio.

Supplemental Figure 1. Patient inclusion flowchart

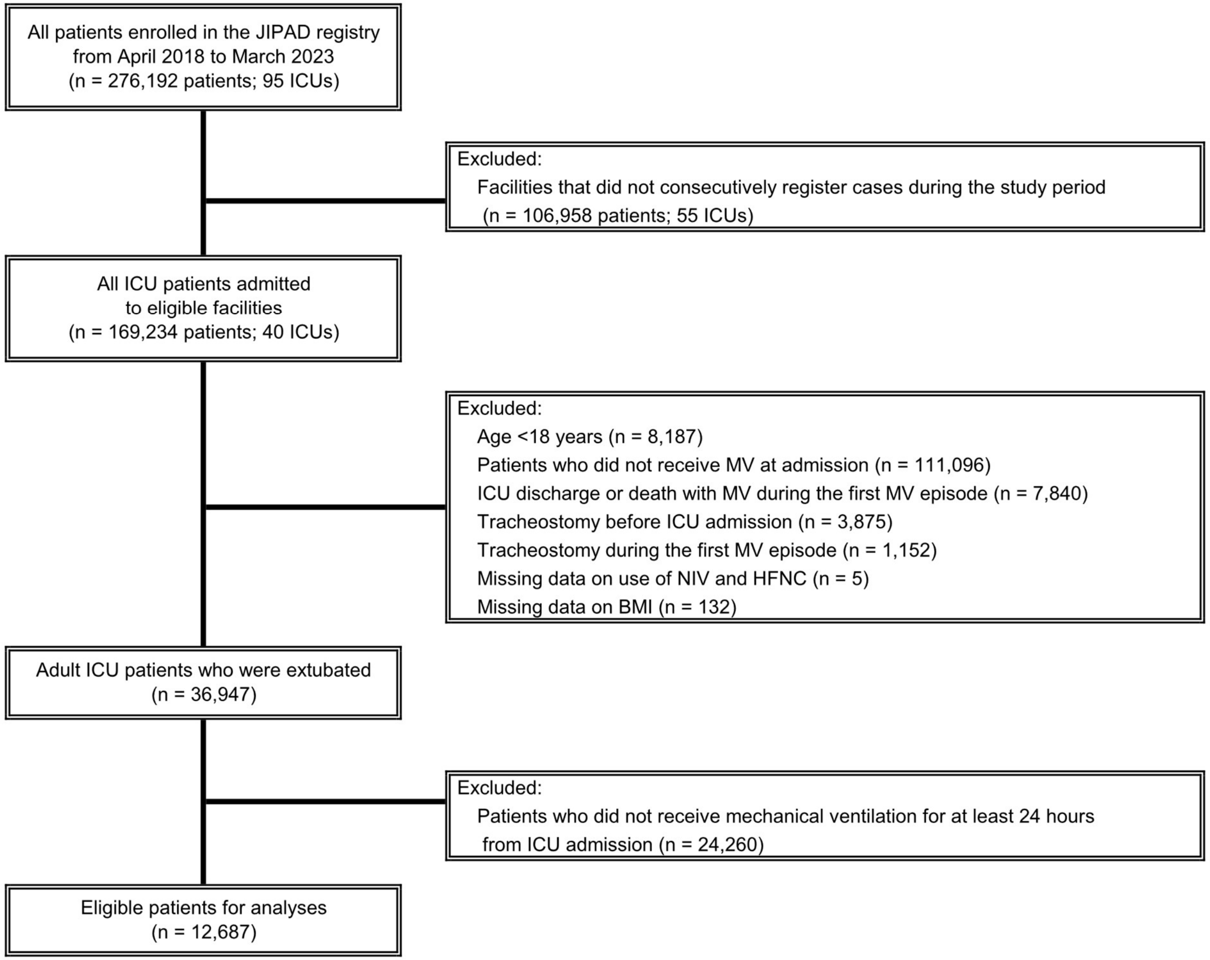

BMI, body mass index; HFNC, high-flow nasal cannula; ICU, intensive care unit; JIPAD, Japanese Intensive care PAtient Database; MV, mechanical ventilation; NIV, non-invasive ventilation.

Supplemental Figure 2. Distribution of reintubation risk factors and reintubation rate by the number of risk factors

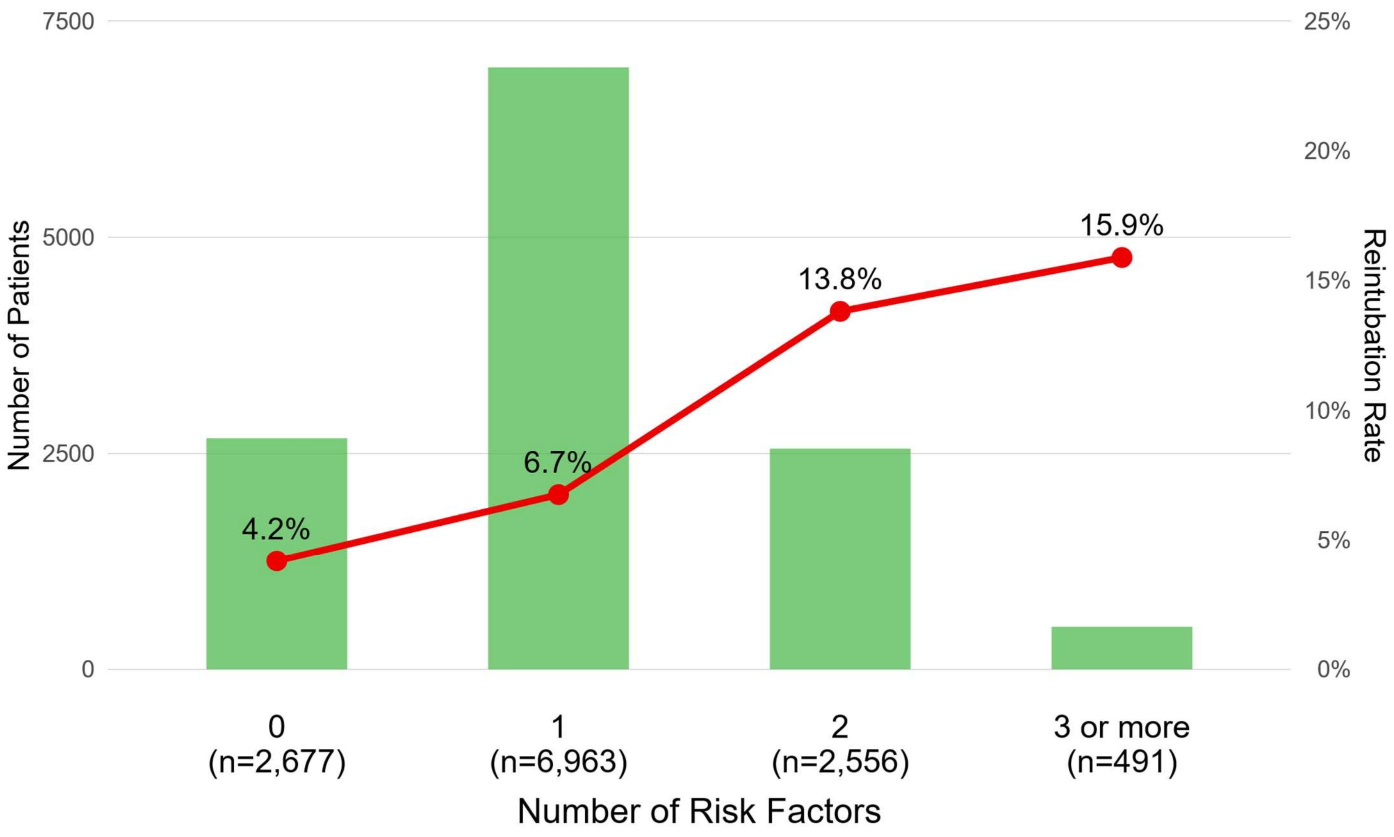

Supplemental Figure 3. Trends in severity scores at ICU admission by fiscal year for each respiratory strategy

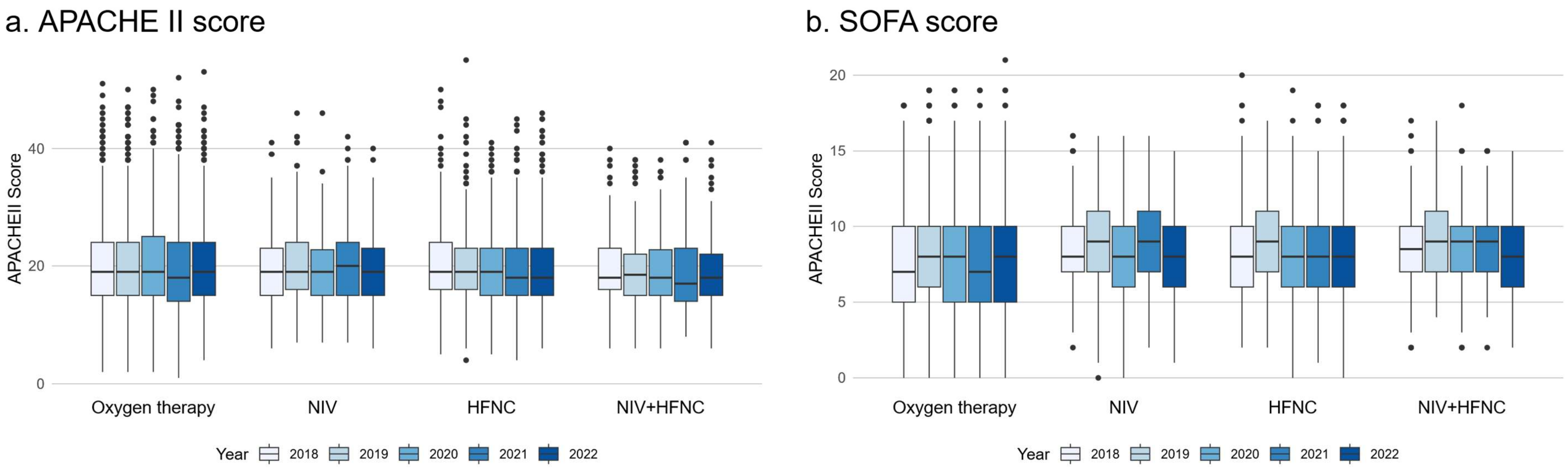

Each fiscal year extended from April 1 to March 31 of the following year.

APACHE, Acute Physiology And Chronic Health Evaluation; HFNC, high-flow nasal cannula; ICU, intensive care unit; NIV, non-invasive ventilation; SOFA, Sequential Organ Failure Assessment.

Supplemental Figure 4. The proportion of each post-extubation respiratory management at each facility

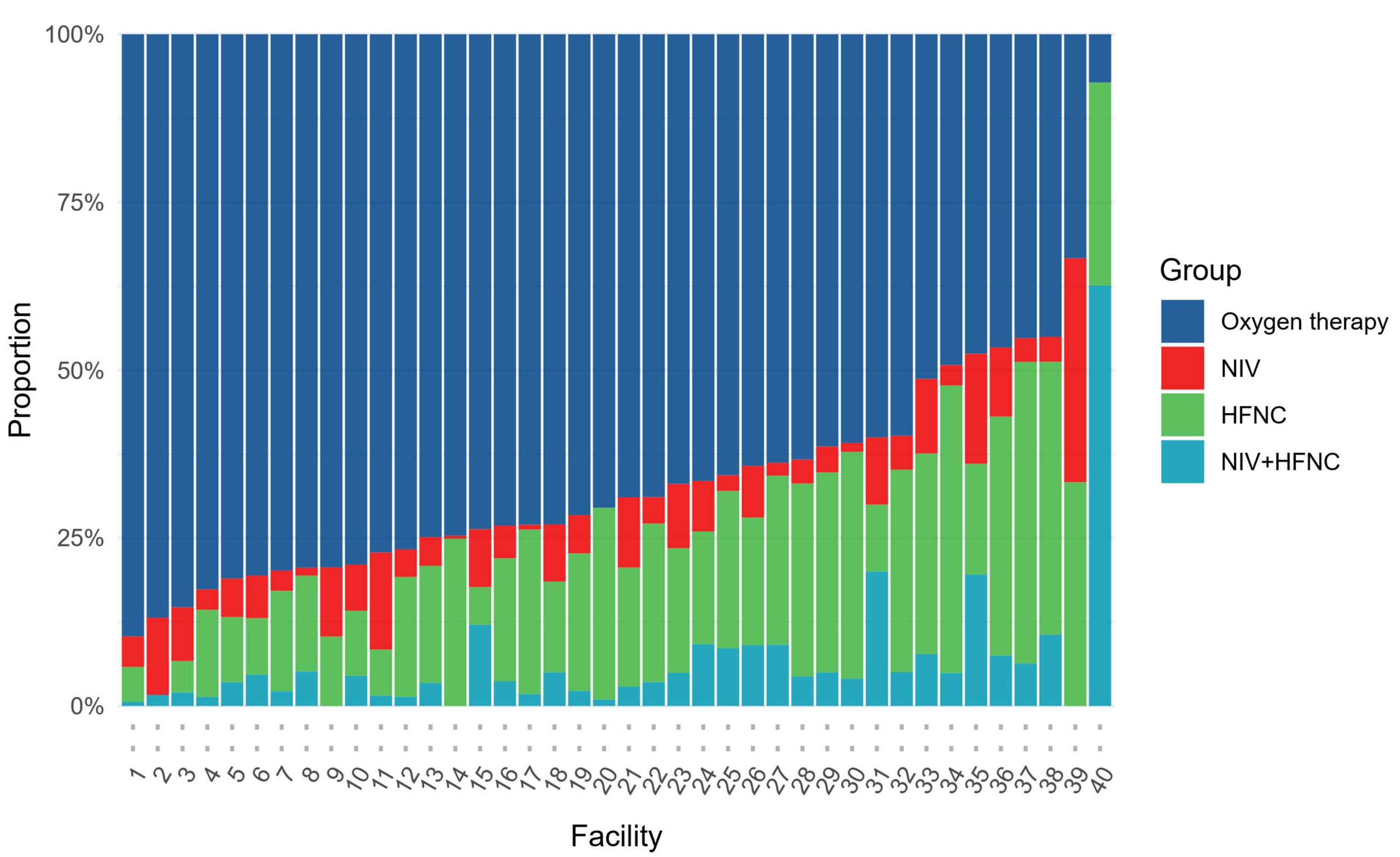

HFNC, high-flow nasal cannula; NIV, non-invasive ventilation.

Supplemental Figure 5. Time from initial extubation to ICU discharge according to the post-extubation respiratory strategy

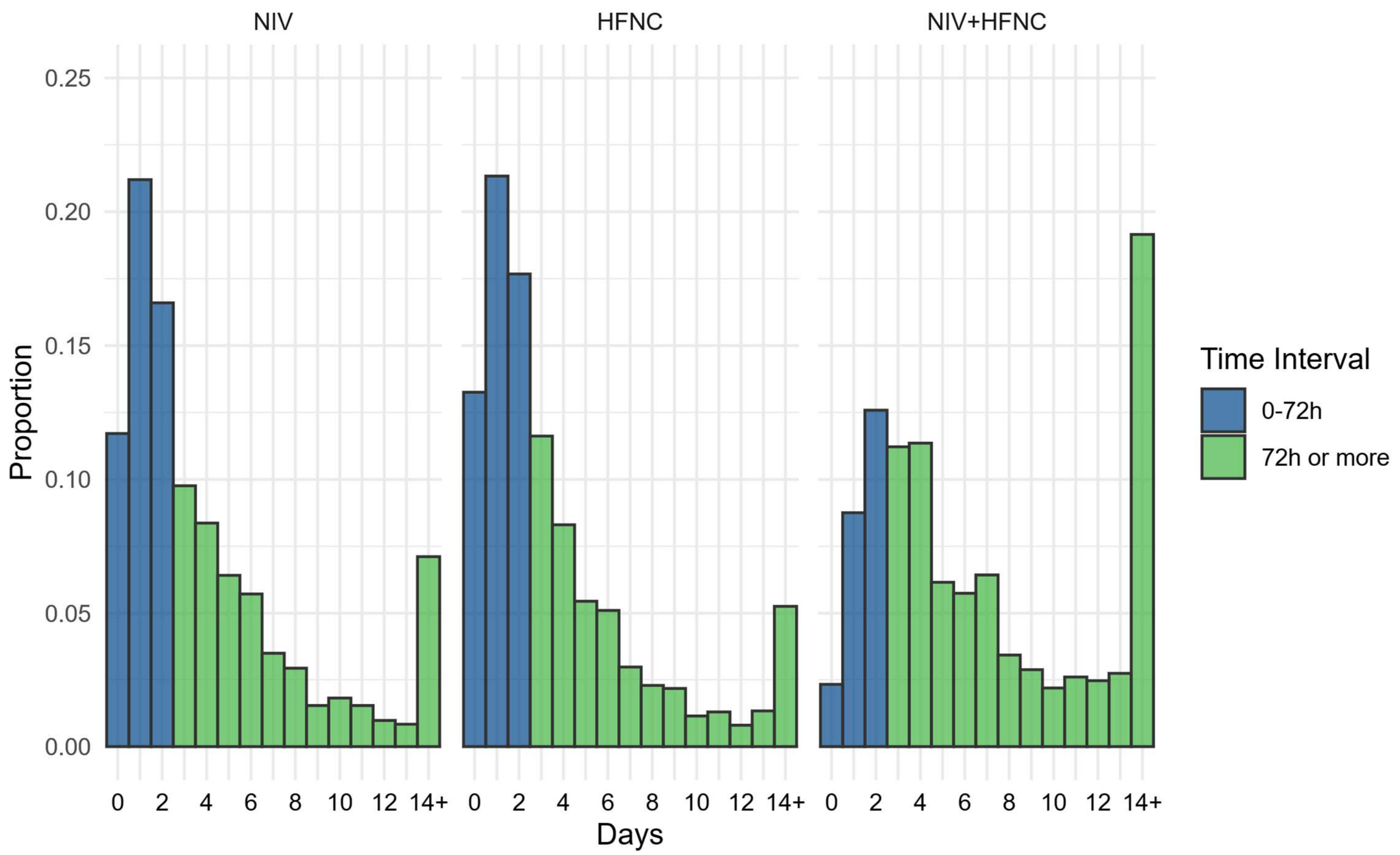

HFNC, high-flow nasal cannula; ICU, intensive care unit; NIV, non-invasive ventilation.

Supplemental Figure 6. Temporal trends in the proportion of respiratory management stratified by subgroups

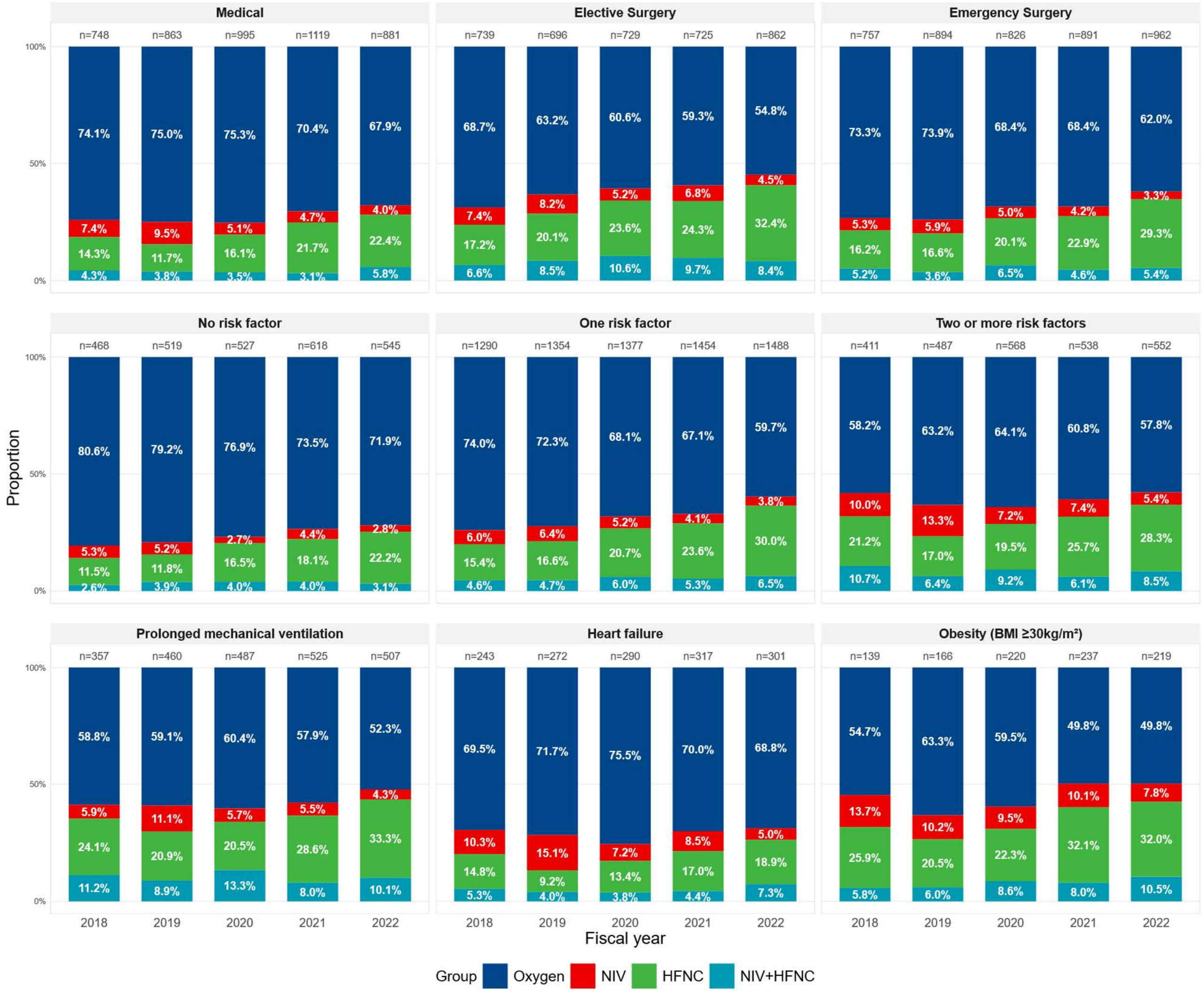

Trends are shown for three subgroup categories: (A) Admission type (medical, elective surgery, and emergency surgery); (B) Number of risk factors (0, 1, and ≥2); and (C) Specific risk factors (obesity, heart failure, and prolonged mechanical ventilation). Each fiscal year extended from April 1 to March 31 of the following year.

HFNC, high-flow nasal cannula; NIV, non-invasive ventilation.
